# The local clinical validation of a new lithium heparin tube with a barrier: BD Vacutainer® Barricor LH Plasma tube

**DOI:** 10.11613/BM.2017.030706

**Published:** 2017-08-28

**Authors:** Fatma Demet Arslan, Inanc Karakoyun, Banu Isbilen Basok, Merve Zeytinli Aksit, Anil Baysoy, Yasemin Kilic Ozturk, Yusuf Adnan Guclu, Can Duman

**Affiliations:** 1 Department of Medical Biochemistry, University of Health Sciences, Tepecik Education and Research Hospital, Izmir, Turkey; 2 Palliative Care Unit, University of Health Sciences, Tepecik Education and Research Hospital, Izmir, Turkey

**Keywords:** serum, plasma, blood analysis, blood specimen collection, evaluation

## Abstract

**Introduction:**

Although serum-providing blood tubes with a barrier are still widely used due to their significant advantages, the use of blood tubes with a barrier to provide plasma is becoming widespread. We compared 22 analytes in a BD Vacutainer® Barricor LH Plasma tube for local clinical validation of this new lithium heparin tube with a barrier.

**Materials and methods:**

Samples from 44 volunteers were collected in different tubes (Becton Dickinson and Company): Z tube without additive (reference), clot-activator tube with gel (SST), lithium heparin tube without gel (LiH), and lithium heparin tube with barrier (Barricor). Analyte concentrations in different tubes were compared with the reference tube. All tubes were also evaluated according to additional testing (different centrifugation durations, blood-sampling techniques and individual differences).

**Results:**

Aspartate aminotransferase (AST), glucose (Glc), potassium (K), lactate dehydrogenase (LD), sodium (Na), and total protein (TP) had a significant bias in Barricor (9.19%, - 3.24%, - 4.88%, 21.60%, - 0.40%, 5.03%, respectively) relative to the reference tube. There was no statistical difference between different centrifugation durations and individual differences for AST, K and LD in LiH and/or Barricor (P > 0.05). There was a significant bias for LD between LiH and Barricor in terms of blood-sampling techniques (21.2% and 12.4%, respectively).

**Conclusions:**

Recently, the use of plasma has become prominent due to some of its advantages. In this study, plasma AST, K, LD, Glc and TP levels in Barricor were clinically different in comparison to serum. The results of additional tests showed that higher levels of LD in Barricor did not result from haemolysis, and they might be related to other factors including number of platelets, cellular fragility, or functional environment.

## Introduction

Serum is the most commonly used specimen for the analysis of biochemical parameters. The use of blood tubes with a barrier or gel has become popularised due to the reduced need to transfer to a secondary tube. Other important advantages that make the use of these tubes so widespread are the lower need for aliquot tubes, improved analytical stability, and reduced haemolysis rates by facilitating rapid separation of serum from the cellular constituents of blood (*1*).

Nevertheless, the use of plasma has important advantages for laboratory professionals: (i) no need for the extra time that is required for blood coagulation; (ii) shorter centrifugation duration; (iii) reduced ‘turnaround time’ (TAT); and (iv) no presence of interference induced by microfibrin. Moreover, in proportion to serum, 15 – 20% more plasma can be obtained from whole blood, and the risk of thrombolysis and haemolysis in plasma is lower than in serum. This means that because the blood cells remain intact in the healthy donors’ plasma, no pseudohyperkalemia occurs in plasma in contrast to serum (*2*). However, as a consequence of not having a tube with an effective barrier, cell-supernatant contact is still a problem for plasma samples. To solve this problem, the plasma samples may be transferred to a secondary tube, but that is a major obstacle to widespread use.

The World Health Organization reports that plasma reflects the pathological condition of the patient better than serum (*3*). Thrombocytes and coagulation factors are activated when the needle is inserted into the vein during blood sampling, and this activation continues in the blood that is collected into anticoagulant-free blood sample tubes. In order to obtain a valid clinical laboratory result, it is necessary to protect the *in vivo* conditions of the components after the body fluid has been separated. By using blood tubes containing anticoagulants, undesirable variations might be prevented (*3*). The recommended anticoagulant is lithium heparin because it presents least disadvantages in obtaining plasma.

Despite the widespread use of clot-activated tubes containing gel, debate continues about the effects of tube selection on clinical results, and simultaneously, some disadvantages are mentioned (*4*–*6*). During biochemical and therapeutic drug analysis, absorption of analytes into gel may occur, and this condition could explain the following by the chemical structure of the gel: the analyte itself, the duration in the gel, the storage temperature and the volume of the sample. The importance of this issue is the fact that the release of gel material into the sample may affect test results (*6*).

The Clinical and Laboratory Standards Institute (CLSI) published the GP-34A guideline in 2010, which detailed procedures for the validation and verification of blood collection tubes (*7*). In fact, this document is essentially orientated to the validation of blood collection tubes in terms of the manufacturers’ perspective in order to ensure that design and performance goals are met. With the awareness that a gap remains between manufacturers validation and clinical laboratories implementation, the Working Group for Preanalytical Phase (WG-PRE) of the European Federation of Clinical Chemistry and Laboratory Medicine (EFLM) has prepared the consensus document with a number of easy-to-apply criteria for laboratory specialists to verify whether new blood collection tubes meet the basic criteria of technical and clinical acceptability to allow them to be used in clinical laboratories (*8*).

At present, a large number of blood collection tubes are available on the market. While the goal of the manufacturers is to produce tubes that can give the most accurate and reliable results cost effectively by matching clinical laboratory demands, laboratory professionals are in search of determination and usage of tubes that will provide the most accurate and reliable results for daily practice. A new blood tube has been recently launched: the Barricor LH Plasma tube, which contains lithium heparin with a specially designed barrier. The tube consists of two components: an elastomer top, which stretches under centrifugation and creates a seal on the inside wall of the tube at the end of centrifugation, and a high-density base, which uses the differential buoyancy between plasma and cells to ensure the separator orientates correctly under centrifugation as stated in the product information. The use of the aforementioned tube, which also provides lithium plasma, could make a difference to laboratory routine by providing an effective separation between cells and supernatant with its barrier. However, the authors have not encountered a study in which those tubes have been evaluated in the literature.

In this study, a total of 22 different analytes, including routine biochemical and immunochemical parameters were analysed in a group of blood collection tubes, which included a clot-activator tube containing gel, a lithium heparinized tube without any barrier, and a newly produced lithium heparinized tube with a barrier, Barricor, by comparing them with glass tubes. Therefore, our aim was to make a local clinical validation of Barricor tube and to test whether there was a need to modify reference ranges whenever these tubes were used. Furthermore, the aim was to evaluate the risks of thrombolysis, haemolysis and cellular fragility in Barricor due to the presence of a barrier; additional tests were also carried out under different conditions in terms of centrifugation duration, blood sampling techniques and individual differences which were expressed as being healthy or not.

## Materials and methods

### Subjects

A total of 44 inpatients in the Palliative Care Unit of the University of Health Sciences, Tepecik Education and Research Hospital and randomly selected healthy volunteers were included in the study in October 2016.

Individuals who had not been referred to the hospital for any reason in the last 6 months, who did not have any complaints or chronic disease, and who did not receive treatment were defined as the healthy group (N = 20). Randomly selected individuals with different biochemical values in the palliative care unit were included in the study as the patient group (N = 24). All volunteers were aged between 18 and 65 and had no pregnancy status. During blood collection from the volunteers, there were no exclusion criteria except inability to find a suitable vein for blood collection, deterioration of vascular integrity, and tourniquet application time longer than 1 minute.

After overnight fasting (8 – 10 hours), blood samples were collected from healthy volunteers between the hours of 08:00 and 10:00 am. Smoking and the consumption of tea or coffee were forbidden from midnight until blood collection. Alcohol consumption was not allowed for 3 days prior to blood sampling. Healthy volunteers were seated in an upright position 1 minute before venipuncture and during the procedure. In the patient group, no fasting condition was requested and the blood was collected in the supine position between the hours of 08:00 and 10:00 am. The blood was collected from the antecubital vein into blood collection tubes using a needle (Becton Dickinson and Company Vacutainer ®Eclipse, blood collection needle, 21Gx 1–1/4”(0.8 x 32 mm), catalogue number 368609, NJ, USA) with each participant. In healthy subjects, the EFLM opinion paper was followed by taking blood samples from two different venipuncture sites (*8*). However, in palliative care patients, blood samples could only be collected from one arm (venipuncture site) due to treatment given in the opposite arm. The blood sampling was performed by a single phlebotomist. Venous blood samples of inpatients were drawn from the arm that was not receiving any treatment. Detailed information was given to all participants about the study before their participation, and their signed consents were obtained. This comparative analytical study was conducted in accordance with the Helsinki Declaration and with the approval of the local ethics committee (Izmir 9 Eylul University’s Ethics Committee, Decision Date-Number: 6th Oct 2016-2016/26-32).

### Methods

Blood samples from each individual were collected in four different types of tubes: a) an additive- and gel-free glass tube (Becton Dickinson and Company (BD) Vacutainer ®Z tube, 7 mL, 13 x 100 mm, catalogue number 367615, NJ, USA) (Z tube); b) a clot-activator tube containing gel (BD Vacutainer ® SST II Advance tube, 5 mL, 13 x 100 mm, catalogue number 367955, NJ, USA) (SST); c) a barrier-free lithium heparinised tube (BD Vacutainer ®BD Lithium Heparin, 4 mL, 13 x 75 mm, catalogue number 368884, NJ, USA) (LiH); and d) a newly produced lithium heparinised tube with a barrier (BD Vacutainer ®Barricor LH Plasma tube, 3 ml, 13 x 75 mm, catalogue number 365031, NJ, USA) (Barricor).

All tubes were kept in an upright position at room temperature for 30 – 45 minutes. Plasma and serum samples were incubated for a minimum of 30 minutes and a maximum of 45 minutes before centrifugation; hence, all tubes had the same effects upon analytes due to cellular contact. Serum and plasma samples were separated according to manufacturers’ centrifugation recommendations. Whereas the Z, SST and LiH tubes were centrifuged for 10 minutes at 1500xg, the Barricor tube was centrifuged for 3 minutes at 2360xg. The serum and plasma samples were transferred to the secondary tubes to discontinue the cell-supernatant contact in the tubes without a gel or barrier. In case of the presence of a barrier, primary tubes were used during analysis. No visible haemolysis was detected in all serum and plasma samples. Measurement of the analytes in the serum or plasma samples of each individual was performed within a total of 2 hours. Care was taken to ensure that the tube sequences were random during the analysis period.

A total of 22 parameters (such as routine biochemical analytes including albumin (Alb), alanine aminotransferase (ALT), alkaline phosphatase (ALP), aspartate aminotransferase (AST), calcium (Ca), creatinine (CREA), direct bilirubin (BD), gamma-glutamyl transferase (GGT), glucose (Glc), lactate-dehydrogenase (LD), total bilirubin (BT), total protein (TP), urea, and uric acid (UA) concentrations) were measured with spectrophotometric methods. Electrolytes, including chloride (Cl), potassium (K) and sodium (Na), were measured with indirect potentiometry using an AU5800 autoanalyser (Beckman Coulter Inc., CA, USA). Immunochemical analytes, including folate, free triiodothyronine (free T3), free thyroxine (free T4), thyroid stimulating hormone (TSH), and vitamin B12 (B12) concentrations, were analysed by chemiluminescence assay method using a DxI immunoanalyser (Beckman Coulter Inc., CA, USA). All analyses were run in duplicate within a single run, and the averages of the results were included. Precision studies were performed with internal quality control materials (QC) (Control Serum, Beckman Coulter Ireland Inc., catalogue number ODC0003, Clare, Ireland; MAS Liquimmune, Microgenics Corporation, catalogue number 10013876, CA, USA) at two different levels per day as part of routine laboratory practice.

Although Barricor has a newly developed tube with a barrier, it still contains lithium heparin. To conclude whether there was a need to change the reference interval or not in any parameter, it was first necessary to verify reference intervals for each analyte. If there was a bias outside the acceptability limits or the reference range, this might indicate a change in the reference range. Therefore, the manufacturer’s recommended plasma reference ranges were used for each test. The reference interval verification was made according to the following guidelines: CLSI GP34-A and CLSI C28–A3 (*7*, *9*). In brief, the reference range transfer was accepted for each tube, if at most 2 (10%) of the test results of each tube obtained from healthy subjects (N = 20) were out of the reference range recommended by the manufacturer.

Further studies were planned to understand the effects of different centrifugation durations, blood collection techniques, and individual differences on some analytes. In order to assess the effects of the centrifugation, blood samples from another 10 apparently healthy individuals were collected in two Barricor tubes. The hypothesis was that a longer centrifugation time might lead to haemolysis. To test that, two different time points were chosen at 3 and 10 minutes. One of the tubes was centrifuged at 2360xg for 3 minutes and the other for 10 minutes under the same conditions. Haemolysis index values were obtained from an AU5800 autoanalyser.

To determine the effects of blood sampling techniques, blood samples from another 10 apparently healthy individuals were collected in Barricor and LiH tubes with the help of both a needle-syringe system and a vacuum extraction system from both arms. Plasma-free haemoglobin (Hb) concentrations were measured using the spectrophotometric method (*10*), and haemolysis indexes were obtained from an AU5800 autoanalyser. The levels of AST, K and LD in both Barricor and LiH tubes were evaluated in terms of different blood collection techniques.

The individual differences were expressed as being healthy or not. To understand whether individual differences had an effect on any analyte or not, 44 subjects (consisting of diseased (N = 24) and apparently healthy individuals (N = 20) in whom initial comparisons had been undertaken) were further included in the study to separately evaluate the bias between the reference tube and the Barricor tube for AST, K and LD parameters.

### Statistical analyses

The Z tube was identified as the reference tube because this tube was an additive- and gel-free glass tube and the other plastic blood tubes might lead to an interference with the test results. This modality was also adopted from a study published by Bowen *et al.* (*5*).

The SPSS 20.0 programme (SPSS Inc. Chicago, USA) was used for all statistical analyses. The normality of the variables was tested with the Shapiro–Wilk test. Continuous variables were presented as mean with standard error of mean (SEM) or median with interquartile range (IQR). If data complied with normal distribution, the statistical difference between the results of the samples was evaluated by the paired t test, and if not, by the Wilcoxon test. While comparing three blood tubes with the reference tube, the Bonferroni method was used to adjust the value of the significance level, and P value < 0.017 was considered as statistically significant. In secondary studies, which were planned to understand the effects of different centrifugation durations, blood collection techniques and individual differences on these analytes, P value < 0.05 was considered as statistically significant.

Whether the analyte concentrations produced clinically significant differences or not was assessed using the ‘significant change method’ (*4*, *5*, *11*). In brief, the usual standard deviation (USD) of 6 months’ internal quality control data for each analyte were collected. The significant change limit (SCL) was calculated as the average of the reference tube results of ± 2.8 USD. A clinically significant difference was defined if the average analyte concentration in each tube exceeded the SCL limit. The bias between the compared tube and the reference tube results has been calculated with the formula as follows: [(average of compared tube results – average of reference tube results) / average of reference tube results] x 100. The desirable quality specifications for bias was determined according to the biological variation data (*12*, *13*). If the bias was higher than the desirable quality specifications for bias based on the biological variation, it was considered as a clinically significant difference. Clinically significant results obtained from the different tubes were also compared by Passing and Bablok regression analyses and subsequently, these results were visually demonstrated on Bland and Altman plots.

## Results

The measurement ranges for each analyte are provided at Appendix 1. The average of results for each analyte determined in different tubes, the SCLs, the desirable quality specifications for bias, the bias values and the statistical significances in comparison to the reference tube are shown in Table 1. Alb, Cl, CREA, urea, free T3, free T4 and B12 concentrations in three different tubes were statistically not different according to the reference tube. A statistically significant difference was detected for BD, LD, Na and TP in the SST tube. The levels of ALP, AST, ALT, Ca, folate, GGT, Glc, K, LD, Na, BT, TSH, TP and UA were statistically different in the lithium heparinised plasma (LiH and/or Barricor) in comparison with serum obtained from the reference tube (Table 1).

**Table 1:** Analytical and clinical data on 22 analytes tested in 3 different types of blood collection tubes in comparison to the reference tube

**Analytes tested**	**Clinical significance limits**			**Z tube**	**SST**			**LiH**			**Barricor**		
**-SCL**	**+SCL**	**Bias_d_**	**Mean** **(SEM)**	**Mean** **(SEM)**	**%Bias**	**P value**	**Mean** **(SEM)**	**%Bias**	**P value**	**Mean** **(SEM)**	**%Bias**	**P value**
**Alb, g/L** **(N = 44)**	36.8	43.7	1.43	40.2 (1.1)	40.2 (1.1)	- 0.14	0.880	40.2 (1.1)	- 0.14	0.447	40.3 (1.1)	0.11	0.466
**ALP, U/L** (N = 43)*	89	119	6.72	105 (13)	104 (13)	- 0.46	0.061	101 (13)	- 3.17	< 0.001	100 (12)	- 3.92	< 0.001
**ALT, U/L** (N = 43)*	21	27	11.48	25 (3)	25 (3)	0.09	0.681	25 (3)	0.56	0.333	25 (3)	4.05	0.006
**AST, U/L** **(N = 44)**	21	30	6.54	25 (3)	26 (3)	1.93	0.022	25 (2)	- 0.45	0.614	28 (3)	9.19	< 0.001
**B12, pg/mL** **(N = 44)**	268	374	12.00	321 (35)	324 (35)	0.92	0.861	319 (34)	- 0.78	0.334	321 (35)	- 0.07	0.933
**BD, µmol/L** **(N = 44)**	-0.76	4.47	14.20	1.86 (0.17)	1.79 (0.17)	- 3.77	0.005	1.93 (0.17)	4.08	0.060	1.90 (0.17)	2.30	0.591
**BT, µmol/L** **(N = 44)**	7.39	12.08	8.95	9.74 (0.51)	10.05 (0.51)	3.19	0.083	10.07 (0.51)	3.39	0.019	10.16 (0.51)	4.39	0.001
**Ca, mmol/L** **(N = 44)**	2.32	2.47	0.82	2.40 (0.02)	2.40 (0.02)	- 0.05	0.880	2.42 (0.02)	0.54	0.018	2.40 (0.02)	- 0.61	0.007
**Cl, mmol/L** **(N = 44)**	97	109	0.50	103 (0.0)	103 (0.0)	- 0.24	0.043	103 (0.0)	- 0.06	0.606	103 (0.0)	- 0.17	0.134
**CREA, µmol/L** (N = 40)*	73	110	3.96	91 (3)	91 (3)	0.04	0.763	91 (3)	- 0.18	0.564	91 (3)	0.27	0.581
**Folate, ng/mL** **(N = 44)**	6.7	8.4	19.20	7.6 (0.7)	7.6 (0.7)	0.84	0.911	7.5 (0.7)	- 0.95	0.264	7.4 (0.7)	- 2.30	0.004
**free T3, pmol/L** **(N = 44)**	3.99	5.83	4.80	4.91 (0.15)	4.90 (0.14)	- 0.16	0.824	4.89 (0.14)	- 0.40	0.629	4.89 (0.14)	- 0.63	0.395
**free T4, pmol/L** **(N = 44)**	10.18	13.31	3.30	11.74 (0.26)	11.83 (0.26)	0.75	0.203	11.73 (0.26)	- 0.12	0.893	11.87 (0.26)	1.07	0.091
**GGT, U/L** (N = 41)*	38	46	6.70	43 (7)	43 (7)	0.14	0.347	42 (6)	- 2.20	< 0.001	44 (6)	5.51	0.183
**Glc, mmol/L** **(N = 44)**	6.0	6.6	2.34	6.3 (0.5)	6.3 (0.5)	0.03	0.683	6.3 (0.5)	0.62	0.006	6.1 (0.5)	***- 3.24***	< 0.001
**K, mmol/L** **(N = 44)**	4.1	4.5	1.81	4.3 (0.1)	4.4 (0.1)	1.05	0.074	**4.0** **(0.1)**	- 7.05	< 0.001	4.1 (0.1)	***- 4.88***	< 0.001
**LD, U/L** **(N = 44)**	170	207	4.30	188 (9)	205 (9)	8.85	< 0.001	175 (8)	- 6.99	< 0.001	229* (*10*)	***21.60***	< 0.001
**Na, mmol/L** **(N = 44)**	133	150	0.23	142 (0)	140 (0)	- 0.75	< 0.001	141 (0)	- 0.55	< 0.001	141 (1)	***- 0.40***	0.012
**TP, g/L** (N = 43)*	67.1	74.7	1.36	71.0 (0.9)	71.2 (0.9)	0.34	0.013	74.6 (0.9)	5.18	< 0.001	74.5 (0.9)	***5.03***	< 0.001
**TSH, mIU/L** (N = 42)*	1.44	1.55	9.70	1.51 (0.15)	1.50 (0.15)	0.71	0.879	1.54 (0.16)	1.96	0.003	1.52 (0.16)	0.83	0.103
**UA, µmol/L** (N = 41)*	266	374	4.87	315 (15)	315 (15)	- 0.54	0.232	322 (15)	1.55	< 0.001	313 (15)	1.03	0.281
**Urea, mmol/L** **(N = 44)**	12.8	15.0	5.57	13.9 (1.1)	13.9 (1.1)	- 0.20	0.278	13.6 (1.1)	- 0.50	0.096	13.9 (1.1)	- 0.26	0.290
(±) SCL – (upper/lower) significant change limit. Bias_d_ - desirable quality specifications for bias. SEM - standard error of the mean. %Bias - difference between the compared tube results and the reference tube results. Z-tube - glass tube without additive (reference tube). SST - clot-activator tube with gel. LiH - lithium heparin tube without gel. Barricor - lithium heparin tube with barrier. Alb, Albumin; ALP, alkaline phosphatase; ALT, alanine aminotransferase; AST, aspartate aminotransferase; B12, vitamin B12; BD, Bilirubin, direct; BT, Bilirubin, total; Ca, calcium; Cl, chloride; CREA, creatinine; free T3, free triiodothyronine; free T4, free thyroxine; GGT, gamma glutamyl transferase; Glc, glucose; K, potassium; LD, lactate dehydrogenase; Na, sodium; TP, total protein; TSH, thyroid stimulating hormone; UA, uric acid. P value was defined as statistical significance of results between the compared tube and the reference tube by using paired t test or Wilcoxon test. P value < 0.017 was considered statistically significant. Values exceeding the significant change limit are presented in bold. Values exceeding desirable quality specifications are presented in bold-italic. *N was less than 44 due to insufficient sample volume.													

When assessed for SCL, no analyte tested in the SST tube remained outside of the limit. However, K in LiH and LD in Barricor were outside the limits. When assessed according to the desirable quality specifications for bias, the bias value of serum Na (- 0.75%) and LD (8.85%) in SST exceeded the desirable limit. The bias values of K (- 7.05%), LD (- 6.99%), Na (- 0.55%) and TP (5.18%) in LiH, and AST (9.19%), Glc (- 3.24%), K (- 4.88%), LD (21.60%), Na (- 0.40%) and TP (5.03%) in Barricor were found to be not acceptable compared to the reference tube. Clinically significant results (AST, K, LD, Glc, TP and Na) obtained from the different tubes were shown using Passing and Bablok regression graphs and Bland and Altman plots in Appendix 2. Although AST, K, LD, Glc and Na results were strongly correlated as seen in the regression analyses, AST and K in the SST tube, Glc in the LiH tube and Na in the Barricor tube had limited constant errors. According to the Bland Altman plots, most paired data lay within confidence interval of agreement limits, except AST and LD in SST, and LD in Barricor in comparison to the Z tube.

Among the parameters analysed, LD, K and TP levels in plasma samples produced clinically important results (Table 1). The bias value (9.19%) for AST in Barricor plasma was also clinically significant. It was possible to speculate that these increases in AST, LD and K concentrations, especially in Barricor plasma, might be due to haemolysis. The difference in TP concentrations between plasma and serum samples was attributed to the presence of fibrinogen in plasma, so further investigation was found unnecessary.

In assessment of the centrifugation, no significant differences were found between the two Barricor tubes in terms of AST, K and LD results according to different centrifugation durations (3 and 10 minutes at 2360 g). The haemolysis index was also negative in all tubes (Table 2).

**Table 2:** The effects of two different centrifugation times on AST, K, and LD in Barricor plasma

**Barricor** **N = 10**	3 minute centrifugation*		10 minute centrifugation*		**P value**
**Median**	**IQR**	**Median**	**IQR**
**AST (U/L)**	23	19 - 30	23	19 - 30	0.687
**K (mmol/L)**	4.3	4.1 - 4.9	4.2	4.1 - 4.8	0.084
**LD (U/L)**	200	188 - 237	210	187 - 239	0.131
Barricor - lithium heparin tube with barrier. IQR - interquartile range. AST, aspartate aminotransferase; K, potassium; LD, lactate dehydrogenase. P < 0.05 was considered statistically significant. *Centrifuged at 2360xg.					

In comparison to blood sampling techniques, there was no significant difference between the two techniques in terms of AST, K, LD and free Hb results in both Barricor and LiH tubes, and the haemolysis index in all tubes was negative. However, in the blood samples collected with the needle-syringe system, a statistically significant difference was found between Barricor and LiH in terms of AST, K, and LD; in the samples collected with the vacuum extraction system, again, significant differences were detected in terms of AST and LD. When the bias between Barricor and LiH tubes for the LD test was evaluated according to the desirable quality specifications for bias (11.4%), it was found to be 21.2% when the blood was collected with the needle-syringe system and 12.4% with the vacuum extraction system, and both values were outside the desirable limits. As expected, when the blood was collected with the needle-syringe system, the bias was higher (Table 3).

**Table 3:** The effects of two different blood collection techniques on AST, K, LD and free Hb results in Barricor plasma samples

	**N = 10**	**Blood collected by needle-syringe system**				**Blood collected by vacuum extraction system**					**Comparison of blood collection methods (P)**
**Mean**	**Median (IQR)**	**% Bias**	**P value**	**Mean**	**Median (IQR)**	**% Bias**	**P value**	**Bias_d_**
**AST** **(U/L)**	**Barricor**	24	26 (20 – 28)	5.6	0.031	24	26 (23 - 27)	7.0	0.004	6.54	0.944
	**LiH**	23	25 (21 – 26)			23	24 (21 - 25)				0.129
**K** **(mmol/L)**	**Barricor**	4.0	4.1 (3.9 - 4.4)	0.7	0.046	3.9	3.9 (3.7 - 4.2)	- 1.7	0.414	1.81	0.221
	**LiH**	4.0	4.0 (3.9 - 4.4)			4.0	4.0 (3.7 - 4.3)				0.799
**LD** **(U/L)**	**Barricor**	189	181 (175 – 214)	21.2	0.007	178	178 (168 - 186)	12.4	0.005	4.30	0.185
	**LiH**	156	155 (142 – 172)			158	158 (147 - 168)				0.767
**free Hb** **(g/L)**	**Barricor**	0.87	0.37 (0.11 - 1.46)	NC	0.953	0.17	0.13 (0.06 - 0.20)	NC	0.153	ND	0.139
	**LiH**	0.76	0.44 (0.10 - 1.65)			0.25	0.22 (0.18 - 0.33)				0.169
IQR - interquartile range. %Bias - difference between Barricor tube results and LiH tube results. Bias_d_ - desirable quality specifications for bias. NC - not calculated. ND - not determined. AST, aspartate aminotransferase; K, potassium; LD, lactate dehydrogenase; free Hb, free haemoglobin. P < 0.05 was considered statistically significant.											

The effects of individual differences were evaluated. Although there was no statistically significant difference between patients and healthy individuals for AST, K and LD in the Barricor plasma samples, the bias between the Barricor and Z tube was observed to be higher in patients than in healthy individuals. When the bias for LD was evaluated according to the desirable quality specifications for bias (4.3%), its value was found to be 25.3% in patients and 16.3% in the healthy individuals, which were also outside of the limits. As the bias for K was assessed according to the desirable quality specifications for bias (1.81%), for healthy individuals K was also outside desirable quality specifications for bias, although lower than for the patients (Table 4).

**Table 4:** The effects of individual differences on AST, K, and LD in Barricor plasma

	**Patients (N = 24)**			**Healthy individuals (N = 20)**			**P**
**Barricor**	**Mean**	**Median (IQR)**	**% Bias**	**Mean**	**Median (IQR)**	**% Bias**
**AST**	30	25 (21 - 28)	10.6	26	23 (20 - 32)	7.4	0.715
**K**	4.0	4.1 (3.7 - 4.2)	- 5.8	4.2	4.2 (3.9 - 4.4)	- 3.8	0.053
**LD**	240	223 (185 - 265)	25.3	218	209 (175 - 254)	16.3	0.429
Barricor - lithium heparin tube with barrier. IQR - interquartile range. %Bias - difference between Barricor and Z tube’ test results in patients and healthy individuals. AST, aspartate aminotransferase; K, potassium; LD, lactate dehydrogenase. P < 0.05 was considered statistically significant.							

## Discussion

As far as we know, this was the first study in which a large number of biochemical analytes have been evaluated in blood sampling tubes that included a newly developed lithium heparinized tube with a barrier, namely, Barricor. AST, K, LD, Glc and TP concentrations in Barricor plasma were clinically different in comparison to serum. To understand the role of the barrier in these results, secondary tests were performed and the findings showed that higher activities of AST and LD in Barricor plasma did not result from haemolysis, and it might be related to the number of platelets, cellular fragility, or the functional environment.

It is reasonable to think that higher TP concentrations in plasma samples than in serum samples might depend on the preservation of fibrinogen, which is an extra protein in the plasma. Indeed, plasma fibrinogen concentrations, defined as 2.0 – 4.0 g/L, were in agreement with the difference. Other studies in the literature also have similar TP results in comparison to serum *vs*. plasma samples (*14*–*16*).

In non-separated blood samples, Glc is still metabolised at approximately 5% to 7% per hour at room temperature because upstream enzymes continue to metabolise it to glucose-6-phosphate (*17*). The clinically significant lower concentrations of Glc only in Barricor plasma might depend on either the barrier not being able to separate plasma from cell compartments, or cellular activity persisting in plasma. The preservation of Glc in LiH plasma might be explained with the transfer of plasma into a secondary tube. While there was no difference between plasma and serum samples in some studies (*14*, *16*, *18*), Ladenson *et al.* found plasma Glc to be significantly lower (5.1%) than serum concentrations (*15*). This was attributed to the presence of erythrocytes and leukocytes in those studies.

It is a known fact that plasma Na concentrations are lower than that of serum, either with or without a barrier (*14*, *19*). Higher serum Na concentrations might be secondary to the release of Na, as platelets underwent viscous metamorphosis. Interestingly, lower concentrations of Na in SST serum were observed in comparison to serum samples from the Z tube and plasma samples from the Barricor and LiH tubes. Nevertheless, the effect of gel separator in SST upon the Na analyte is not understood and remains a matter to be investigated.

In this study, AST in Barricor plasma had a higher bias than AST in LiH plasma. Doumas *et al.* found a similar elevated bias and hence proposed that the presence and concentration of heparin might lead to it (*16*). Nevertheless, in this study the concentrations of heparin in Barricor and LiH tubes were not different. Therefore, it is possible to postulate that bias in Barricor might be due to the turbulence effect of the barrier and/or prolonged cell contact.

Potassium concentrations in barrier-free plasma samples were found to be lower than in either serum or Barricor plasma samples. As emphasised in the literature, it is already expected that K is higher in serum due to the excretion from erythrocytes and thrombocytes during the coagulation process (*14*–*16*, *18*, *19*). It is also stated that this phenomenon may be related to individual characteristics such as thrombocyte concentrations or thrombocyte function (*15*). In fact, that is exactly why plasma is the recommended sample type for K measurement (*14*–*16*, *18*, *19*). However, higher concentrations of K in the tubes with a barrier than in the barrier-free LiH tubes may be due to prolonged cell contact, but further investigation is needed to explain the underlying cause. In a recent study on sample stability, the basal K concentrations in Barricor plasma were lower than the K concentrations in Plasma Separator (PST) plasma in contrast to these findings (*20*). Therefore, it is necessary to evaluate these Barricor tubes in different settings to clarify the net effects.

In the Barricor plasma, LD activities were clinically different compared to serum samples and plasma in LiH. Indeed, serum is the recommended matrix for LD analysis as reported in the literature (*21*–*24*). Even manufacturers recommend serum matrix for LD activity measurement. As seen in this study, LD activity is higher in plasma than in serum, and the higher activities of LD might be explained with cellular contamination (*14*–*16*, *18*, *19*). Herzum *et al.* have stated that, although there were very high thrombocytes in the separated plasma, the contamination had little effect on LD (*25*). In support of this opinion, Bakker and colleagues have proved that LD errors were not related to sample preparation in order to achieve cell elimination from the plasma (*26*). According to the study published by Møller *et al.,* the centrifugation had an effect on LD results by increasing it to 6.3% (*27*). Other factors could explain this discrepancy between the serum and plasma LD results such as testing method, blood collection devices and sample transport apart from cellular contamination and sample preparation. However, unlike in LiH plasma, higher LD was found in the Barricor plasma in this study. No information about the possible mechanism underlying this difference has been detected in the literature, possibly due to the fact that Barricor is a new generation blood sampling tube containing lithium heparin with a barrier. Higher LD activities either for SST or Barricor in the presence of a separator (gel or barrier) were notable. A possible cellular contact, and hence contamination, might be prevented in a very short period because there was a transfer procedure into the secondary tubes in those tubes without a separator. In the case of tubes containing a separator, LD may have increased due to the prolonged contact time or the continuation of cellular shift, despite the presence of a separator. In any case, a further explanation for the increase in LD is worth investigating in a prospective study.

The hypothesis that plasma AST, K and LD activities in the LiH tube being found to be lower than for plasma in the Barricor tube could be due to the presence of a possible haemolytic effect in the Barricor tube was further investigated. It was reasonable to think that since the position of the barrier is very close to the tube lid level in the newly produced Barricor tube, the haemolysis resulting from the turbulence as a consequence of the collision and disintegration of cells during blood sampling might be the reason for elevated AST, K and LD results. To prove the hypothesis, further testing was carried out with the Barricor tubes for AST, K and LD under different conditions, which could lead to the haemolysis effect, such as centrifugation durations, different blood sampling techniques and individual differences. Neither plasma haemolysis index nor plasma-free Hb concentrations were different between the barrier-free LiH tube and the Barricor tube. Accordingly, the different centrifugation durations and the blood collection techniques did not have any impact on these tests. However, even with both being used to obtain plasma samples, the difference in LD between Barricor and LiH tubes remained clinically significant. The bias for patient subjects (25.3%) has been observed as being higher compared to healthy subjects (16.3%) in blood samples collected in the Barricor tube. This difference for the patients has been attributed to cellular fragility. All these results indicate that LD elevation may be related to the number of platelets, cellular fragility and the functional environment, besides haemolysis.

There are also some limitations in this work. EFLM opinion was followed by taking blood samples from two different venipuncture sites in healthy subjects (*9*). However, in palliative care patients, blood samples could only be collected from one arm (venipuncture site) due to treatment given in the opposite arm. The haemolysis index and free Hb activities were measured, but the number of erythrocytes and platelets remaining in plasma or serum matrices could not have been evaluated at the cellular base. Furthermore, due to the large number of parameters that were analysed in limited serum volumes, it was not possible to evaluate haemolysis by using the haemolysis index of an autoanalyser in the initial experiments and hence, haemolysis was only visually evaluated in these studies. Only tubes from one manufacturer were evaluated, and also only on one analytical platform. Testing a limited number of samples in secondary studies including centrifugation, blood collection techniques, and individual differences was also a limitation. Lastly, the glass tube was chosen as a reference tube, but the tube used in practice contains clot activator with a serum separator gel. If the aforementioned tube were to be used as the reference tube, it was observed that the bias levels could have been lower.

In recent years, the use of the plasma matrix has become prominent due to some of its advantages. Although the reference range transfer was confirmed in this study, TP, LD and K concentations of plasma were statistically and clinically different compared to serum. In summary, for each existing blood collection tube, each laboratory must either verify the reference interval transfer or create its own reference interval.

## Supplementary material

Appendix 1. The measurement ranges of all analytes tested

Appendix 2. Passing and Bablok graphs and Bland and Altman plots for aspartate aminotransferase (AST), glucose (Glc), potassium (K), lactate dehydrogenase (LD), sodium (Na) and total protein (TP) analysed in 3 different types of blood collection tubes (Z tube, glass tube without additive (reference tube); SST, clot-activator tube with gel; LiH, lithium heparin tube without gel; Barricor, lithium heparin tube with barrier). Only clinically significant parameters were compared by Passing Bablock regression analysis and also visually demostrated with Bland Altman plots. The solid, dashed, and dotted lines in Passing Bablok regression graphs represent the regression line, its confidence intervals, and the identity line (x=y), respectively. The thick solid, dashed, and thin solid in Bland Altman plots represent the mean difference, the limits of agreement, and confidence intervals of limits of agreement, respectively.
