# Supplementary material for: The local clinical validation of a new lithium heparin tube with a barrier: BD Vacutainer® Barricor LH Plasma tube
Source: Biochem Med (Zagreb). 2017 Aug 28;27(3):030706. doi: 10.11613/BM.2017.030706 (PMC5575652; doi:10.11613/BM.2017.030706)
Supplement: Supplementary file 1 — Appendix 1. The measurement ranges of all analytes tested [file bm-27-3-030706-S1.pdf]

Appendix 1. The measurement ranges of all analytes tested

	Tubes	N	Minumum	Maximum
**Alb, g/L**	Z tube	44	19.5	49.0
	LiH	44	20.5	52.0
	SST	44	20.0	49.0
	Barricor	44	20.5	49.5
**ALP, U/L**	Z tube	44	39	626
	LiH	44	38	590
	SST	44	39	622
	Barricor	43	39	581
**ALT, U/L**	Z tube	44	2	95
	LiH	44	3	94
	SST	44	3	98
	Barricor	43	2	93
**AST, U/L**	Z tube	44	8	122
	LiH	44	8	119
	SST	44	8	122
	Barricor	44	10	126
**B12, pg/mL**	Z tube	44	150	1500
	LiH	44	145	1500
	SST	44	139	1500
	Barricor	44	138	1500
**BD, µmol/L**	Z tube	44	0.60	8.72
	LiH	44	0.51	7.95
	SST	44	0.86	8.29
	Barricor	44	0.86	7.52
**BT , µmol/L**	Z tube	44	5.13	21.38
	LiH	44	5.13	22.23
	SST	44	5.13	22.23
	Barricor	44	5.13	22.23
**Ca, mmol/L**	Z tube	44	1.9	2.7
	LiH	44	2.0	2.6
	SST	44	1.9	2.7
	Barricor	44	2.0	2.6
**Cl, mmol/L**	Z tube	44	94.0	109.5
	LiH	44	94.0	110.5
	SST	44	93.5	111.0
	Barricor	44	94.0	109.5
**CREA, µmol/L**	Z tube	43	66.3	172.4
	LiH	44	66.3	172.4
	SST	44	70.7	176.8
	Barricor	41	61.2	172.4
**Folate, ng/dL**	Z tube	44	3.2	24.2
	LiH	44	3.0	24.2
	SST	44	3.3	24.2
	Barricor	44	3.0	24.2

	Tubes	N	Minumum	Maximum
**free T3, pmol/L**	Z tube	44	2.06	7.22
	LiH	44	1.99	7.00
	SST	44	1.98	7.02
	Barricor	44	2.07	6.85
**free T4, pmol/L**	Z tube	44	9.00	17.87
	LiH	44	8.84	17.36
	SST	44	9.32	17.29
	Barricor	44	8.95	18.17
**GGT, U/L**	Z tube	44	9	244
	LiH	44	9	234
	SST	44	9	249
	Barricor	41	9	237
**Glc, mmol/L**	Z tube	44	3.7	22.5
	LiH	44	3.8	22.0
	SST	44	3.8	22.3
	Barricor	44	3.6	21.8
**K, mmol/L**	Z tube	44	3.32	4.97
	LiH	44	3.03	4.72
	SST	44	3.32	4.88
	Barricor	44	3.09	4.78
**LD, U/L**	Z tube	44	118	405
	LiH	44	114	375
	SST	44	132	429
	Barricor	44	149	447
**Na, mmol/L**	Z tube	44	135.0	147.5
	LiH	44	134.0	148.0
	SST	44	134.0	148.5
	Barricor	44	134.5	147.0
**TP, g/L**	Z tube	44	50.0	81.0
	LiH	44	55.0	89.0
	SST	44	51.5	79.5
	Barricor	43	55.0	83.5
**TSH, mIU/L**	Z tube	44	0.01	3.63
	LiH	44	0.02	3.79
	SST	43	0.01	3.67
	Barricor	43	0.01	3.58
**UA, µmol/L**	Z tube	43	139	499
	LiH	42	153	502
	SST	43	139	502
	Barricor	41	136	496
**Urea, mmol/L**	Z tube	44	4.8	37.3
	LiH	44	4.8	37.1
	SST	44	5.0	37.5
	Barricor	44	5.0	36.9

Z tube - glass tube without additive (reference tube). SST - clot-activator tube with gel. LiH - lithium heparin tube without gel. Barricor - lithium heparin tube with barrier. Alb, Albumin; ALP, alkaline phosphatase; ALT, alanine aminotransferase; AST, aspartate aminotransferase; B12, vitamin B12; BD, Bilirubin, direct; BT, Bilirubin, total; Ca, calcium; Cl, chloride; CREA, creatinine; free T3, free triiodothyronine; free T4, free thyroxine; GGT, gamma glutamyl transferase; Glc, glucose; K, potassium; LD, lactate dehydrogenase; Na, sodium; TP, total protein; TSH, thyroid stimulating hormone; UA, uric acid.
