# Supplementary material for: The local clinical validation of a new lithium heparin tube with a barrier: BD Vacutainer® Barricor LH Plasma tube
Source: Biochem Med (Zagreb). 2017 Aug 28;27(3):030706. doi: 10.11613/BM.2017.030706 (PMC5575652; doi:10.11613/BM.2017.030706)
Supplement: Supplementary file 2 — Appendix 2. Passing and Bablok graphs and Bland and Altman plots for aspartate aminotransferase (AST), glucose (Glc), potassium (K), lactate dehydrogenase (LD), sodium (Na) and total protein (TP) analysed in 3 different types of blood collection tubes (Z tube, glass tube without additive (reference tube); SST, clot-activator tube with gel; LiH, lithium heparin tube without gel; Barricor, lithium heparin tube with barrier). Only clinically significant parameters were compared by Passing Bablock regression analysis and also visually demostrated with Bland Altman plots. The solid, dashed, and dotted lines in Passing Bablok regression graphs represent the regression line, its confidence intervals, and the identity line (x=y), respectively. The thick solid, dashed, and thin solid in Bland Altman plots represent the mean difference, the limits of agreement, and confidence intervals of limits of agreement, respectively. [file bm-27-3-030706-S2.pdf]

Appendix 2. Passing and Bablok graphs and Bland and Altman plots for aspartate aminotransferase (AST), glucose (Glc), potassium (K), lactate dehydrogenase (LD), sodium (Na) and total protein (TP) analysed in 3 different types of blood collection tubes (Z tube, glass tube without additive (reference tube); SST, clot-activator tube with gel; LiH, lithium heparin tube without gel; Barricor, lithium heparin tube with barrier). Only clinically significant parameters were compared by Passing Bablock regression analysis and also visually demostrated with Bland Altman plots. The solid, dashed, and dotted lines in Passing Bablok regression graphs represent the regression line, its confidence intervals, and the identity line (x=y), respectively. The thick solid, dashed, and thin solid in Bland Altman plots represent the mean difference, the limits of agreement, and confidence intervals of limits of agreement, respectively.

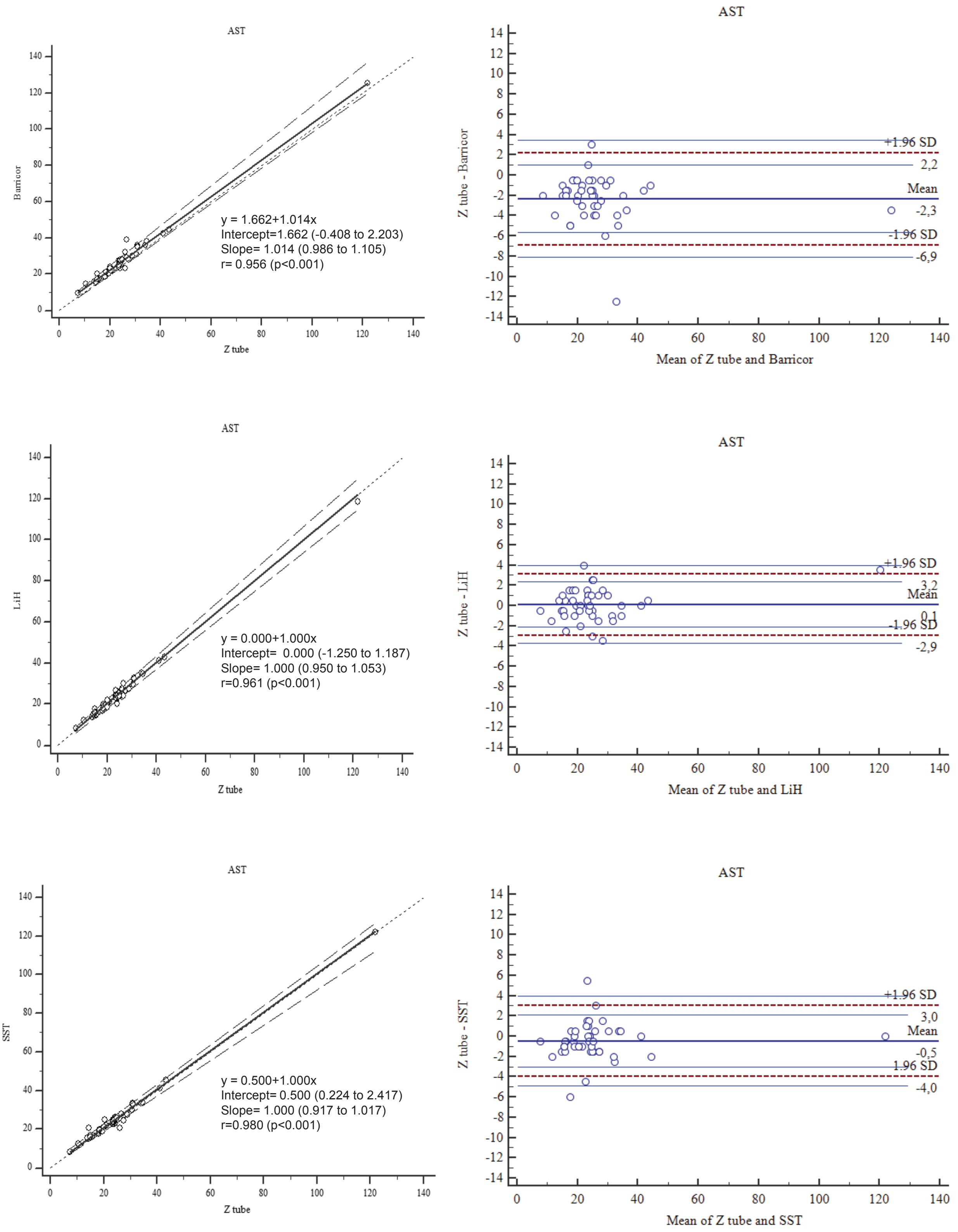

Figure 1. Passing Bablok graphs and Bland Altman plots for AST

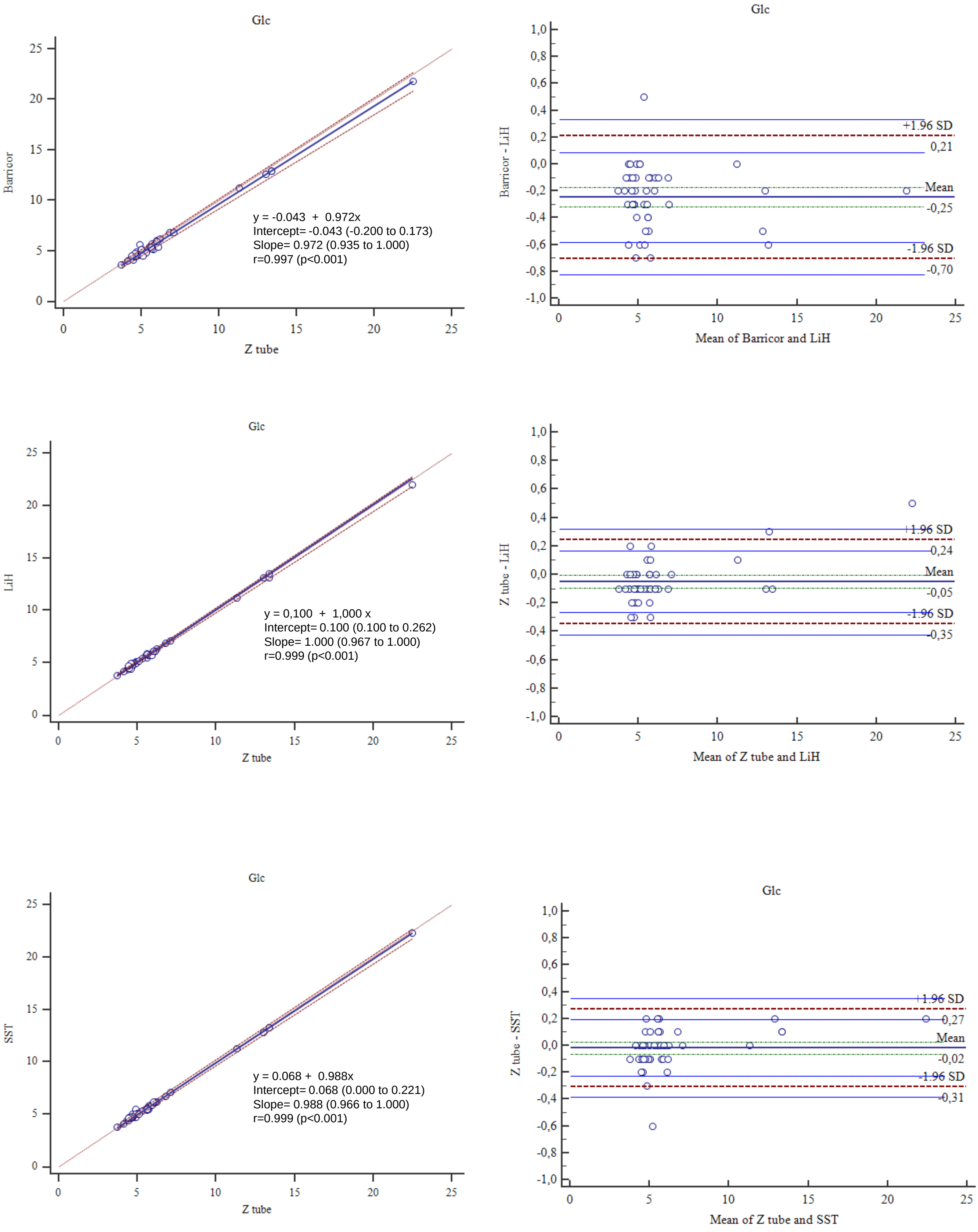

Figure 2. Passing Bablok graphs and Bland Altman plots for Glc.

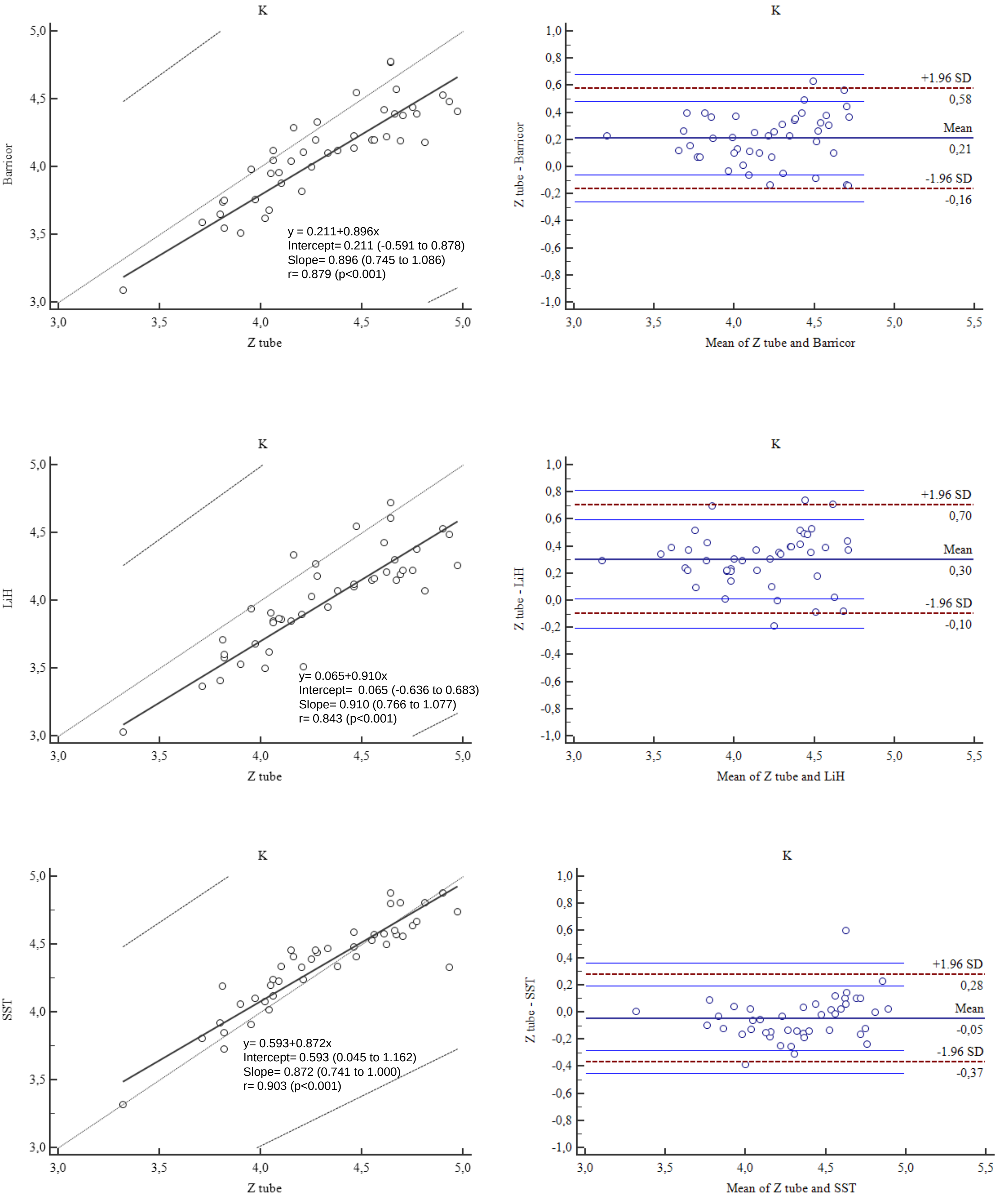

Figure 3. Passing Bablok graphs and Bland Altman plots for K

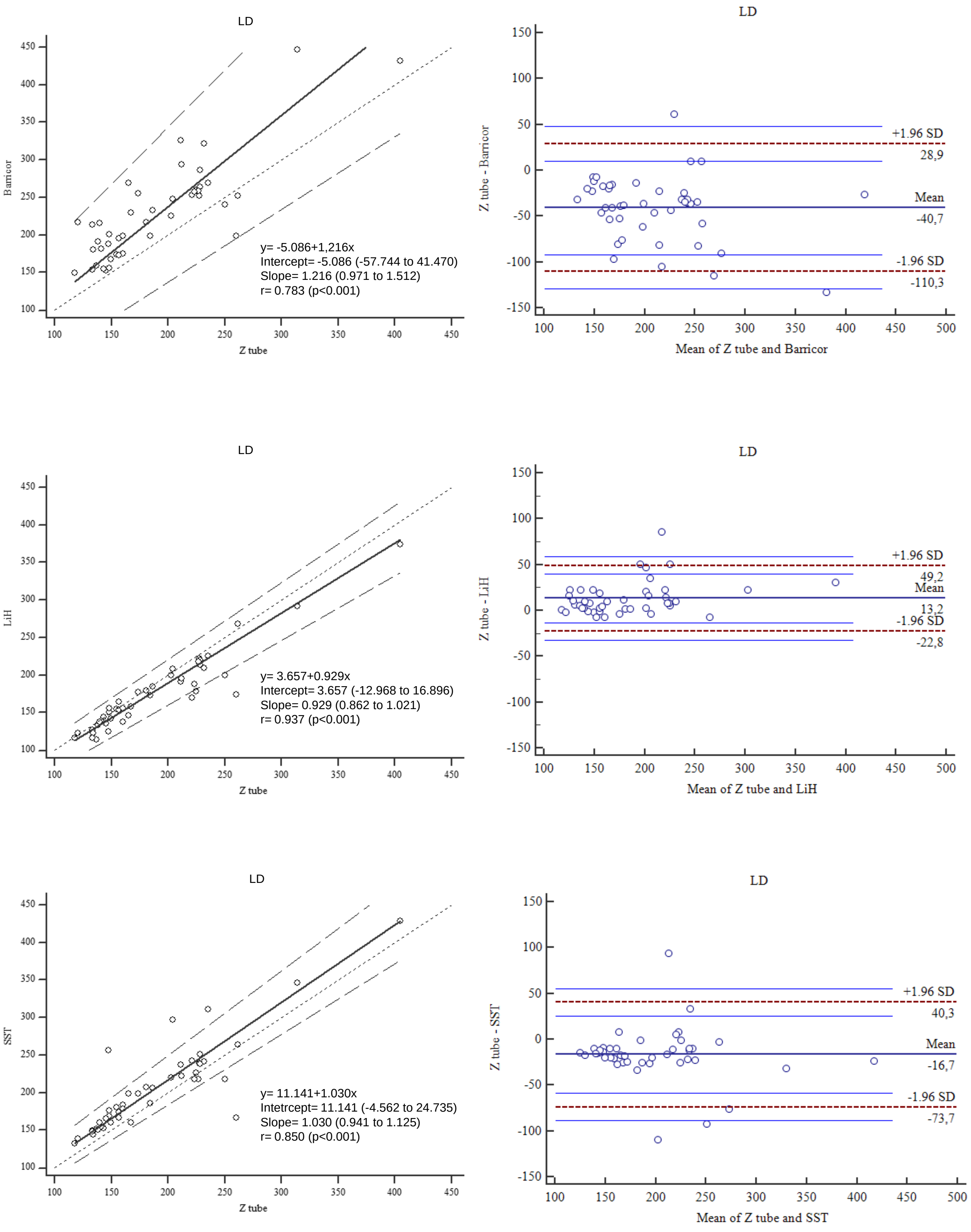

Figure 4. Passing Bablok graphs and Bland Altman plots for LD

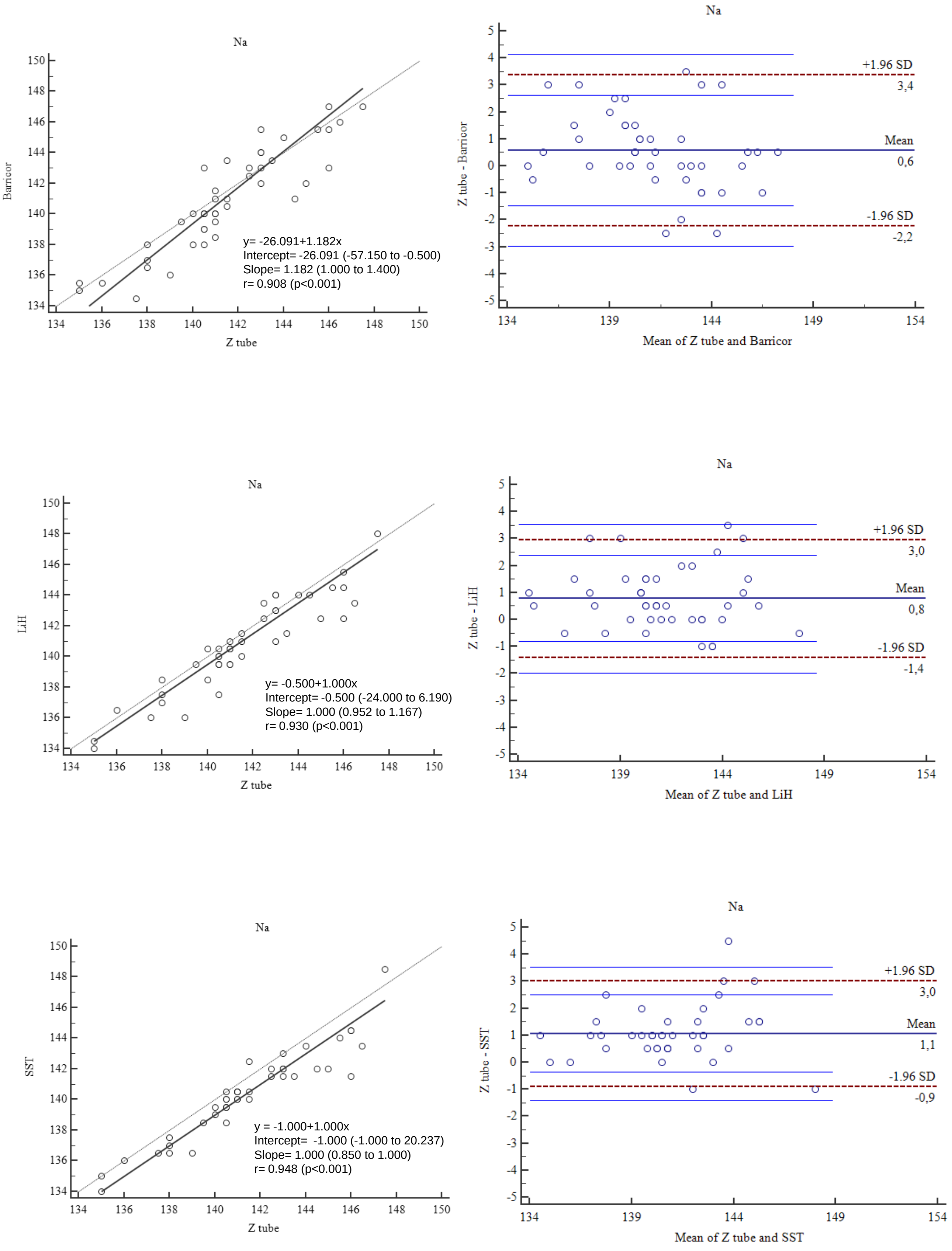

Figure 5. Passing Bablok graphs and Bland Altman plots for Na

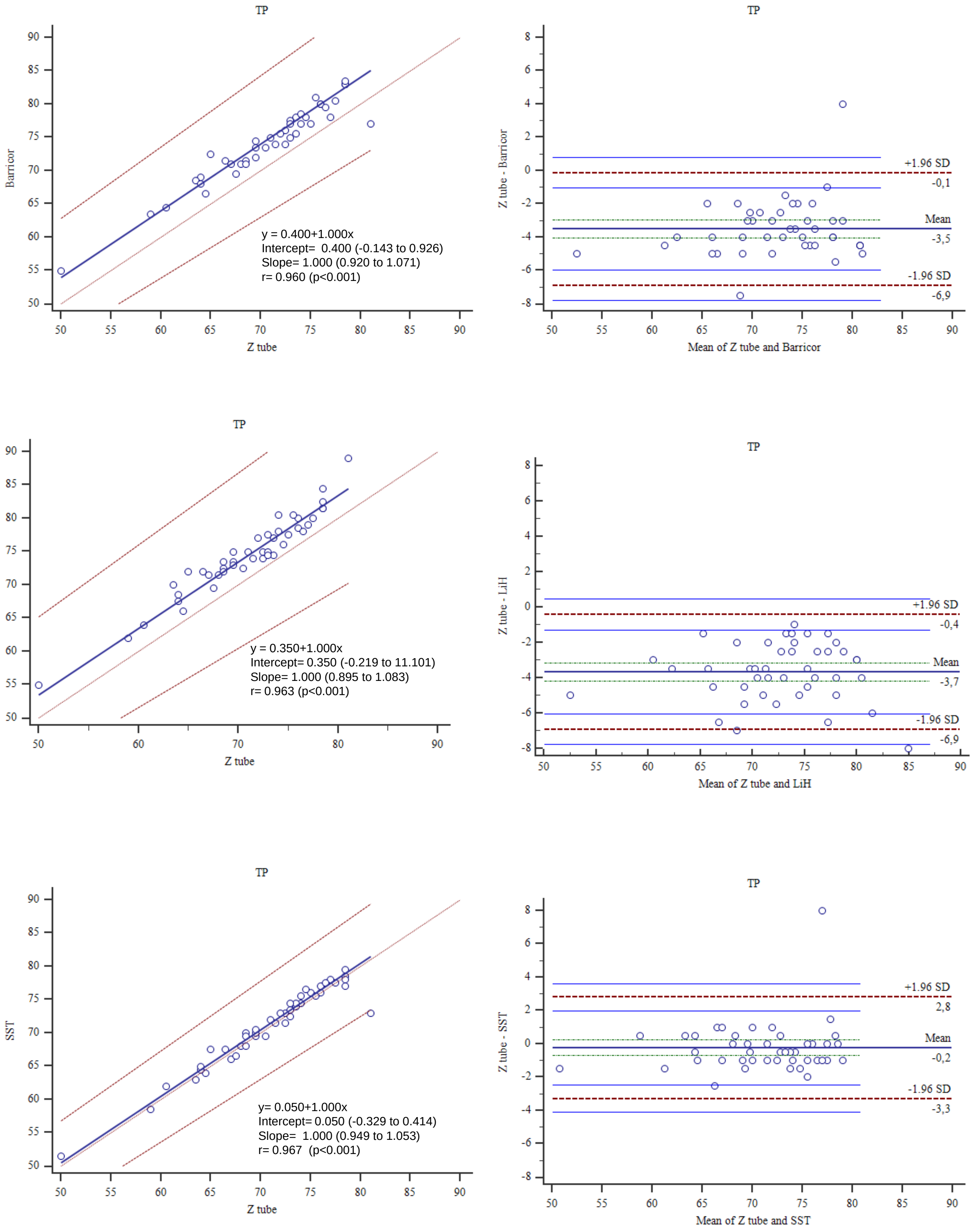

Figure 6. Passing Bablok graphs and Bland Altman plots for TP
